# Supplementary material for: Patterns of Intron Gain and Loss in Fungi
Source: PLoS Biol. 2004 Nov 30;2(12):e422. doi: 10.1371/journal.pbio.0020422 (PMC532390; doi:10.1371/journal.pbio.0020422)
Supplement: Table S1 — Also available at http://genes.mit.edu/NielsenEtAl/. (4.3 MB ZIP). [file pbio.0020422.st001.zip › NielsenEtAl/html/455.html]

AN4522.1.NCU07857.1.MG05296.1.FG04149.1


```
 CLUSTAL W (1.82) Multiple Sequence Alignments - Introns Inserted


Sequence 1: NCU07857.1	117 aa
Sequence 2: MG05296.1	116 aa
Sequence 3: FG04149.1	110 aa
Sequence 4: AN4522.1	116 aa
Alignment Length: 117 aa
Number Identitical Residues: 73 aa
Alignment Score (without introns) 3527


MG05296.1 	MPNNRVT~Y~RRRNP2YNTSSNRTRLIKTPGGDLRILHIKKRGTAPKCGDCGIKLPG0VP
NCU07857.1	MASSRVT~Y~RRRNP2YNTTSNKTRIVKTPGGQLRVLHIKKRGTAPKCGDCGIKLPG0VP
FG04149.1 	MANMRVH2Y~-----~-NTTSNRTRVIKTPGGDIRLLHIKKRGTVPKCGDCGSKLSG0IP
AN4522.1  	MANNRLQ~Y0RRRNP2YNTRSNRVRIVKTPGGELRYQHLKKQGTAPKCGDCGIKLPG0IP
          	*.. *:  *    ..  ** **:.*::*****::*  *:**:**.******* **.* :*

MG05296.1 	ALRPREYAQISKPKKTVQRAYGGSRCGNCVRDRVVRAFLIEEQKIVKKVLKEQTQGDKKK
NCU07857.1	ALRPREYAQLSKPKKTVQRAYGGSRCGNCVRDRVVRAFLIEEQKIVKKVLKEQSQAEKSK
FG04149.1 	ALRPREYSQISKPKKTVQRAYGGSRCGNCVRDRIVRAFLIEEQKIVKKVLKEQEQSQKKK
AN4522.1  	ALRPREYAQISRPKKNVSRAYGGSRCAGCVKDRIVRAFLIEEQKIVKKVLKESQEKAAKR
          	*******:*:*:***.*.********..**:**:******************. :   .:

MG05296.1 	-
NCU07857.1	K
FG04149.1 	-
AN4522.1  	-
          	
```
